# FAK/src-Family Dependent Activation of the Ste20-Like Kinase SLK Is Required for Microtubule-Dependent Focal Adhesion Turnover and Cell Migration

**DOI:** 10.1371/journal.pone.0001868

**Published:** 2008-04-02

**Authors:** Simona Wagner, Chris J. Storbeck, Kristin Roovers, Ziad Y. Chaar, Piotr Kolodziej, Marlene McKay, Luc A. Sabourin

**Affiliations:** 1 Department of Cellular and Molecular Medicine, University of Ottawa, Ottawa, Ontario, Canada; 2 Cancer Therapeutics, Ottawa Health Research Institute, Ottawa, Ontario, Canada; Dresden University of Technology, Germany

## Abstract

Cell migration involves a multitude of signals that converge on cytoskeletal reorganization, essential for development, immune responses and tissue repair. Using knockdown and dominant negative approaches, we show that the microtubule-associated Ste20-like kinase SLK is required for focal adhesion turnover and cell migration downstream of the FAK/c-src complex. Our results show that SLK co-localizes with paxillin, Rac1 and the microtubules at the leading edge of migrating cells and is activated by scratch wounding. SLK activation is dependent on FAK/c-src/MAPK signaling, whereas SLK recruitment to the leading edge is src-dependent but FAK independent. Our results show that SLK represents a novel focal adhesion disassembly signal.

## Introduction

Migration is required for numerous biological processes such as development, tissue repair and regeneration. Signal transduction events governing cell migration involve an ever-expanding number of molecules functioning in interconnected biochemical pathways regulating the turnover of adhesion complexes at the leading edge of migrating cells. Stimulation of cell adhesion and migration induces the formation of integrin-FAK-src complexes required for the recruitment and activation of a number of adaptor molecules leading to focal adhesion turnover and migration [1]–[3]. Indeed, FAK-null cells assemble large and stable adhesion complexes leading to migratory deficits [4]. Similarly, a FAK mutant at tyrosine 397, deficient for c-src binding, fails to induce focal adhesion disassembly in FAK-deficient fibroblasts [5]–[7]. Supporting this, src-family kinase-deficient cells or cells expressing kinase inactive v-src display larger focal adhesions that fail to disassemble [8], [9].

In addition to a an amino-terminal serine/threonine kinase domain, the Ste20-like kinase SLK bears a central coiled-coil domain and a carboxy-terminal AT1-46 [10] homology (ATH) domain [11], [12] of unknown function. Elevated SLK expression and activity leads to rapid actin stress fiber disassembly in a Rac1-dependent manner [13]. We have previously shown that SLK localizes to vinculin-rich ruffles at the cell periphery in spreading fibroblasts, suggesting a role for SLK in adhesion dynamics [13]. Consistent with a role in cytoskeletal rearrangements, SLK has been shown to indirectly associate with the microtubule network [13] and is required for fusion of C2C12 myoblasts into differentiated myotubes [14]. Interestingly, SLK has also been shown to regulate cell cycle progression [15]. In addition, SLK overexpression has been shown to induce an apoptotic response [12]. Supporting a role for SLK in cell death and cellular stress, cleavage of SLK by caspase 3 results in its activation [11]. Similarly, anoxia-recovery also activates a SLK/p38-dependent apoptotic response [16].

Our previous studies showed that SLK overexpression induced a rapid actin stress fiber disassembly that could be partially rescued by co-expression of dominant negative Rac1 [13]. Furthermore, fibroblasts expressing an activated SLK c-terminal truncation failed to assemble large peripheral adhesions during spreading on fibronectin, suggesting that SLK is an important regulator of cytoskeletal dynamics [13]. Here we show that SLK co-localizes with microtubules and adhesion components at the leading edge of migrating cells. We demonstrate that SLK is activated following scratch wounding of fibroblast monolayers in a FAK-src-MAPK-dependent manner. We find that SLK knockdown or expression of a dominant negative version results in impaired microtubule-dependent adhesion turnover and delayed migration. Overall our results show that SLK is a novel regulator of focal adhesion turnover and cell migration.

## Results

### SLK is activated by monolayer wounding and is required for cell migration

We have previously shown that SLK can be co-precipitated with α-tubulin and that it localizes to membrane ruffles at the periphery of spreading fibroblasts [13]. In addition, SLK appears to induce actin stress fiber breakdown through a Rac1-mediated pathway [13]. As the signaling pathways activated during cell spreading also regulate cell motility [1], [2], [17], [18], we tested the possibility that SLK may play a role in cell migration.

**Figure 1:**
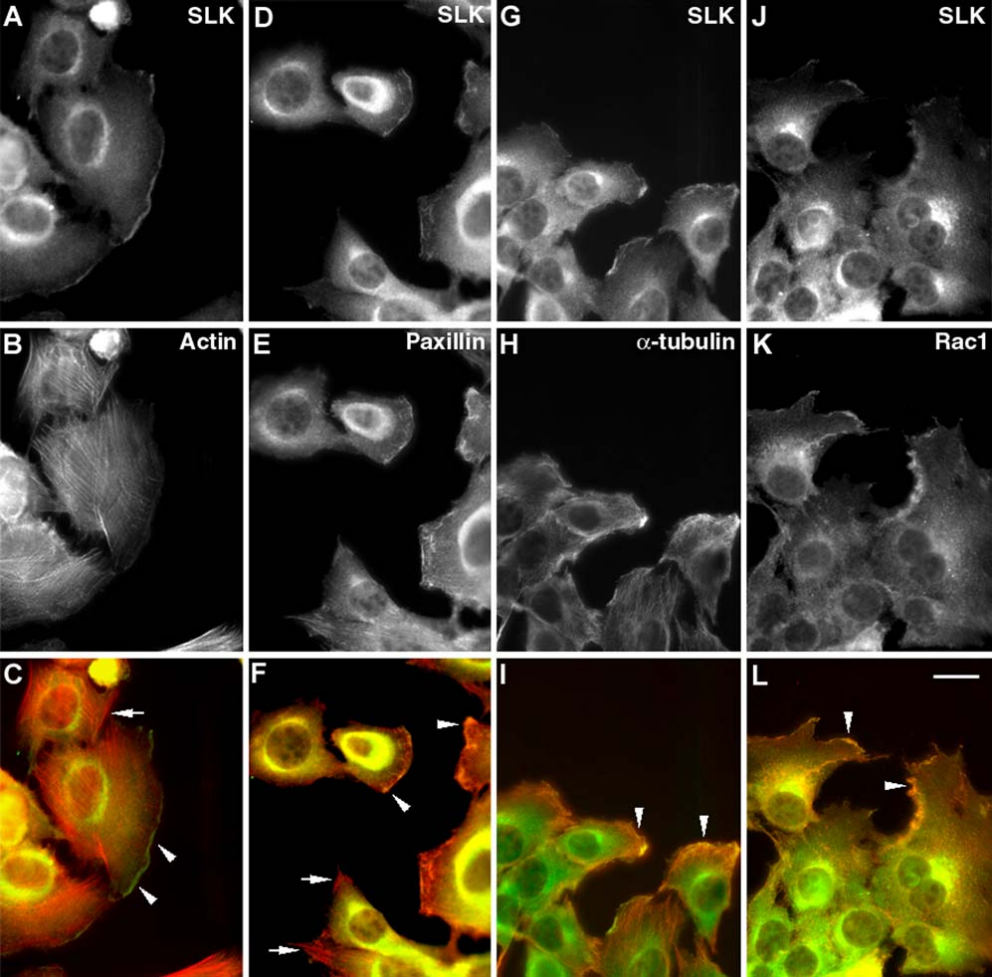
SLK is recruited to the leading edge. MEF 3T3 monolayers on fibronectin-coated coverslips were scratch wounded and allowed to migrate for 2–3 hours. Monolayers were immunostained for SLK in combination with actin (A–C), paxillin (D–F), α-tubulin (G–I), or Rac1 (J–L). In addition to perinuclear staining, SLK was found to be recruited into membrane ruffles (arrowheads) at the leading edge with the other markers surveyed. SLK was not found in mature adhesion complexes as shown by the lack of co-localization between SLK and paxillin in these structures (arrows). All photomicrographs are shown at 400×. Scale bar 10μ.

To test this, we initially investigated the localization of SLK and other cytoskeletal markers following scratch wounding of fibroblast monolayers. Co-immunostaining of SLK with actin stress fibers shows that, in addition to a perinuclear distribution, it is also enriched at the leading edge of migrating cells but not along stress fibers (figure 1A–C). Similarly, at the leading edge, SLK was found to co-localize with paxillin and Rac1 in structures reminiscent of membrane ruffles (figure 1D–F and J–L). Interestingly, it did not localize to large focal adhesions as evidenced by the lack of co-localization with paxillin at these sites (figure 1D–F). Supporting our previous results demonstrating SLK-tubulin co-precipitation [13], SLK co-localized with the microtubule network at the leading edge (figure 1G–I). These observations suggest that SLK is recruited at the leading edge of migrating cells with other adhesion signaling proteins.

**Figure 2:**
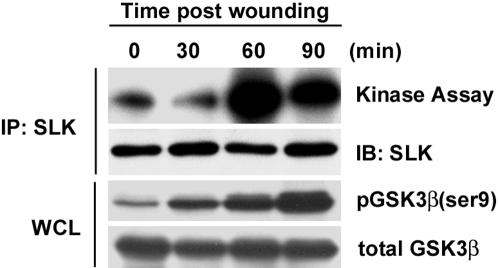
SLK is activated by scratch wounding and is required for cell migration.

Scratch wounding of confluent monolayers has been shown to induce polarization and migration [19], [20]. Therefore, we investigated SLK kinase activity at various time points following scratch wound induced migration of fibroblast monolayers. Immunoprecipitation and *in vitro* kinase assays show that SLK kinase activity is markedly increased following scratch wounding of confluent fibroblasts, with a peak of activity at 60 minutes followed by a decline at 90 minutes (figure 2). Extended time courses up to 120 minutes have shown that SLK activity does not return to basal levels observed at time 0 (not shown). This is likely due to the continued cell migration that occurs following wounding. As previously reported for wounded astrocyte monolayers [20], inactivation of GSK3β also occurred over the time course, indicating polarization and migration of the wounded monolayer. Together, these data indicate that SLK is activated during cell migration and suggest a role for SLK in this process.

**Figure 3:**
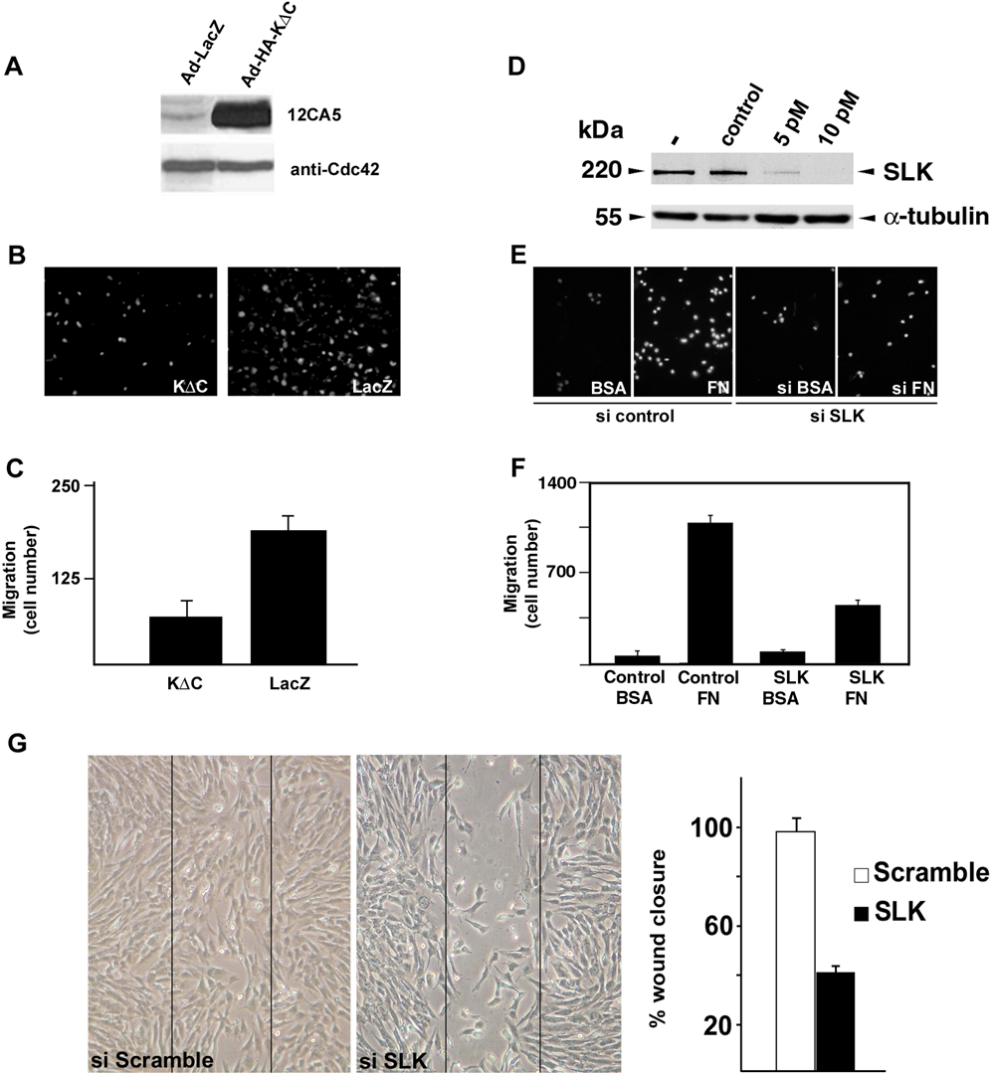
SLK knockdown or expression of a dominant negative SLK inhibits cell migration. Subconfluent MEF 3T3 cells were infected with Adenovirus vectors expressing DN SLK (Ad-HA-KΔC) or LacZ control and subjected to fibronectin (FN) transwell migration assays. (A) Western blot analysis of HA-KΔC expression. Cdc42 was used as a loading control. (B) Polycarbonate membranes were DAPI stained and cells on the underside were enumerated (C) in random fields and expressed as the average/field from triplicate wells. (D) MEF 3T3 cells were transfected with SLK siRNAs and analysed for SLK expression. Western blot analysis of treated lysates indicates that SLK siRNA at 10 pM resulted in a marked knockdown of SLK. Reprobing the membrane with a α-tubulin antibody was used as a control for loading (lower panel). (E–F) Cells were treated with SLK-specific or control siRNAs and assayed for migration through a chamber coated with bovine serum albumin (BSA) (10 µg/ml) (control) or fibronectin (FN) (10 µg/ml). In both cases a 60–70% reduction in migration was observed. (G) Confluent MEF3T3 cells were infected with Adenovirus vectors expressing a scramble or SLK shRNA and manually scratched with a pipet tip. Wound closure was followed for 12 h and the percent closure was evaluated.

To investigate the potential role of SLK in cell migration, MEF-3T3 cells were infected with adenoviral vectors expressing a truncated kinase inactive (DN) form of SLK, SLK^1–373^K63R (HA-KΔC; [13]), or a LacZ control, and subjected to transwell migration assays. Overexpression of DN-SLK in fibroblasts (figure 3A) resulted in a 60–70% inhibition of migration on fibronectin-coated transwell inserts (figure 3B and C). To definitively demonstrate a role for SLK in cell migration, we transfected small interfering RNA (siRNA) molecules specific for murine SLK into MEF-3T3 fibroblasts and assayed their migration in a transwell assay. Following siRNA transfection, the levels of SLK protein were efficiently reduced at 5 pM of siRNA and undetectable at 10 pM compared to control siRNA treated cells (Figure 3D). As for DN-SLK, cells treated with SLK siRNA showed a ∼60% decrease in migration compared to siRNA control treated cells (figure 3E and F). Supporting this, monolayer wounding of shSLK-expressing cells showed a marked delay in wound closure (figure 3G). Together, these data strongly support a role for SLK in the process of cell migration.

### SLK is required for efficient focal adhesion turnover

Efficient cell migration requires focal adhesion turnover at the leading edge of migrating cells [2], [4], [6]–[9], [21]. This process is dependent on the assembly of a functional FAK-src complex initiated by FAK autophosphorylation at tyrosine residue 397 and the recruitment of signaling adapter proteins [1]–[3], [5], [22]–[24]. Interestingly, microtubule disruption leads to stable adhesion complex assembly characterized by high levels of both FAK-Tyr397 [25], [26] and actin stress fibers [26]. Supporting this, stable adhesions contain high levels of phospho-FAK-Tyr397 [5]. Nocodazole wash-out results in focal adhesion turnover and cell migration characterized by cyclical changes in pY397-FAK levels [25]. Because of its association with the microtubule and the requirement for this structure in the process of focal adhesion turnover, we investigated the role of SLK in microtubule-dependent adhesion turnover.

**Figure 4:**
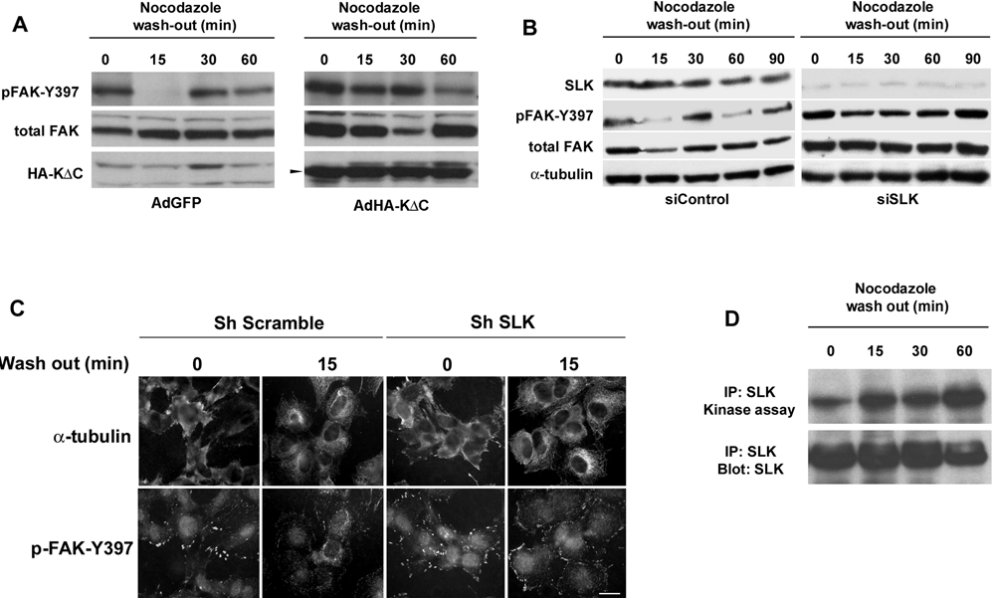
SLK is required for microtubule-dependent adhesion turnover. Subconfluent MEF 3T3 fibroblasts were infected with adenoviral constructs (A) encoding kinase-defective SLK (AdHA-KΔC) or an AdGFP control or transfected (B) with SLK siRNA (or siControl). Cultures were then treated with nocodazole (10 µM) for 4 h, washed and surveyed for FAK-pTyr397 levels over time. Expression of HA-tagged SLK or SLK knockdown was confirmed by Western blot analysis. SLK knockdown or expression of a kinase-deficient SLK interferes with focal adhesion turnover as evidenced by the delayed disappearance of FAK-pTyr397. (C) The status of the microtubule and pFAK-Y-397 was assessed following nocodazole wash-out in the presence or absence of shSLK expression. Large adhesions could still be observed in shSLK expressing cells following wash-out. (D) SLK in vitro kinase assay showing the induction of kinase activity following nocodazole wash-out as described in A and B. Scale bar 10μ.

**Figure 5:**
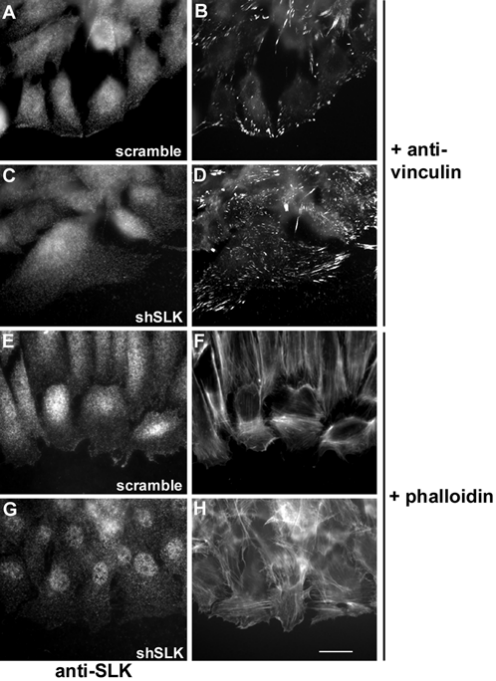
SLK knockdown results in adhesion stabilization. Monolayers of MEF3T3 on FN were infected with adenovirus expressing shSLK or an shScramble control and scratch wounded. After 2 hours, the cells were fixed and stained for SLK (A, C, E and G) in combination with vinculin (B and D) or phalloidin (F and H). In addition to reduced SLK staining, shSLK expressing cells showed no SLK immunoreactivity at the leading edge with an increased number of focal adhesions. No overt differences were observed in phalloidin stained samples. Scale bar 10μ.

MEF-3T3 cells were infected with adenovirus carrying DN-SLK or transfected with SLK siRNAs and subjected to microtubule-dependent focal adhesion turnover assays [25]. As shown in figure 4, following nocodazole wash-out, control treated cultures show the cyclical changes in pY397-FAK. However, in cultures where SLK has been efficiently knocked down, adhesion turnover is severely impaired as evidenced by the sustained levels of pY397-FAK. Similarly, a marked delay in pY397-FAK reduction (15 min vs 60 min) was observed in cultures expressing the DN SLK1-373^K63R^ following nocodazole wash-out. As revealed by pY397-FAK and tubulin immunostaining, nocodazole treated cultures displayed large focal adhesions that disassembled as the microtubule repolymerized (figure 4C; t = 15 min). However, cells expressing a SLK shRNA still displayed enlarged adhesion after the wash-out, suggesting impaired turnover. Interestingly, SLK kinase assays for the same time course showed that its activity was low in cells with large adhesions or high levels of pY397-FAK (figure 4D). Following nocodazole wash out, SLK kinase activity was upregulated within 15 minutes, as focal adhesions disassembled and was upregulated further at t = 60 min, correlating with reduced levels of pY397-FAK. Longer time courses, up to 120 minutes, showed no further change in SLK activity, perhaps due to the fact that adhesion turnover has reached a steady state level. Supporting these observations, siRNA-mediated knock-down of SLK in migrating monolayers (2 hours post-wounding) resulted in an increase in the density and size of vinculin-positive adhesions (figure 5A–D), suggesting that the absence of SLK may result in adhesion stabilization. Interestingly, we could not observe any consistent differences in phalloidin stain following SLK knock-down, suggesting that there are no effects on actin fiber dynamics or that they are very subtle (figure 5E–H). Together, these results suggest that SLK-dependent signals are required to mediate microtubule-dependent focal adhesion turnover.

### Activation and redistribution of SLK requires FAK/src/MAPK signaling

The assembly and activation of a FAK/c-src complex during cell motility appears to be required for efficient focal adhesion turnover [5], [7], [27]. In addition, the activated FAK/c-src complex can recruit multiple signaling adapters and activate downstream signaling through several pathways [2], [3], [28]–[31]. The activation of SLK following scratch wounding of monolayers appears to be a relatively late event (60 min; figure 2), suggesting that upstream signaling may be necessary prior to SLK activation. Because of the ultimate requirement for the FAK-c-src complex in cell migration, we tested whether src family kinases or FAK were required for SLK activation in a scratch wound assay.

**Figure 6:**
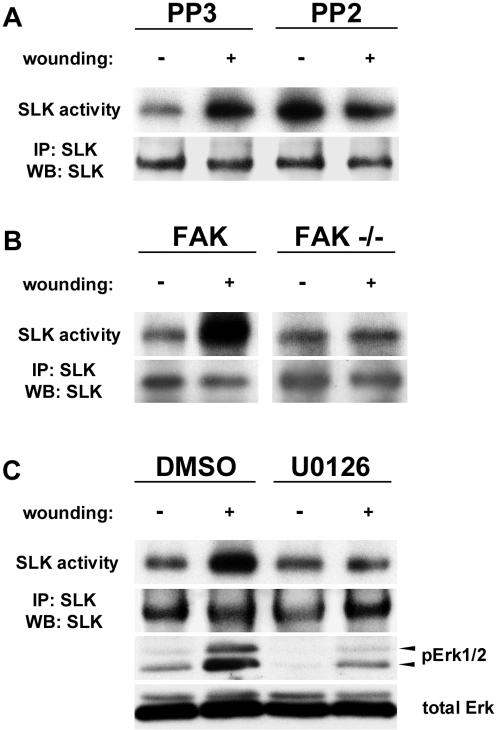
SLK activation requires FAK/src/MAPK signaling. (A) Confluent MEF 3T3 monolayers were pre-incubated (60 min) with inhibitors and then scratch wounded in the presence of inhibitors. Cells were collected 60 minutes later and analysed for SLK kinase activity. (A) Treatment with PP2 or PP3 control. (B) FAK-null or wildtype cells were subjected to scratch wound assays as above and assayed for SLK activity. (C) Treatment with U0126 and DMSO control. Phospho-Erk1/2 is shown as a control for U0126 treatment. SLK activation requires FAK/src/MAPK signaling.

To test the involvement for src family kinases in SLK regulation, monolayers were pretreated with the Src-family inhibitor PP2 (or PP3 control) and then subjected to wounding in the presence of the inhibitor. As shown in figure 6A, treatment with the control PP3 resulted in SLK activation 60 minutes following scratch wounding. Interestingly, treatment with PP2 resulted in an increase in SLK activity in unscratched confluent monolayers that could not be further increased by wounding. Similar results were obtained in SYF cells and rescued when c-src was expressed (not shown). This suggests that src family kinases are required to negatively regulate SLK activity. Supporting this, our previous results have shown that overexpression of v-src inhibits SLK kinase activity in a CKII-dependent manner [32].

To investigate the role of FAK in SLK regulation, scratch wounding assays were performed on FAK(-/-) fibroblasts and wildtype controls. Following wounding of FAK wildtype monolayers, SLK kinase activity is upregulated to levels comparable to that of MEF3T3 cells (figure 6B). However, little or no SLK upregulation was observed following the wounding of FAK-null monolayers (figure 6B), suggesting that adhesion signaling is required and that SLK activation is not a secondary effect of monolayer wounding. Similarly, scratch wounding in the presence of the MEK1 inhibitor U0126 prevented SLK upregulation when compared to a DMSO control (figure 6C). For the same time course, scratch wounding resulted in ERK1/2 phosphorylation whereas it was markedly reduced in the presence of U0126 (figure 6C). Similar experiments in the presence of the p38 inhibitor SB203580 showed no effect on SLK activation (not shown). Overall, these results suggest that SLK activation by scratch-induced motility requires the FAK/c-src/MAPK signaling system.

**Figure 7:**
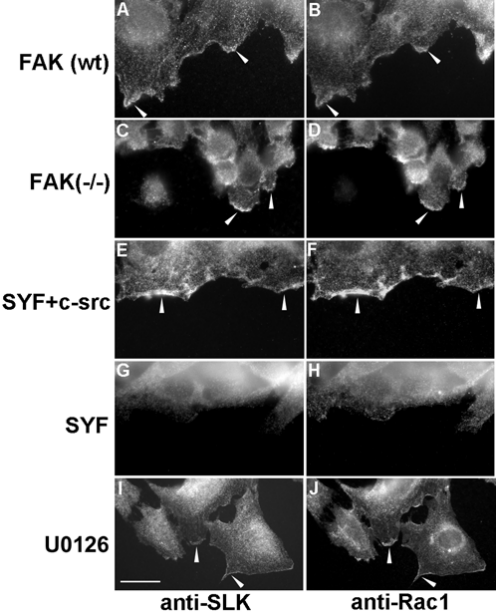
Recruitment of SLK at the leading is c-src-dependent. Confluent monolayers of FAK wildtype (A–B), FAK-null (C–D), SYF +c-src (E–F) and SYF (G–H) cells were scratch wounded and immunostained for SLK and Rac1. Similarly, MEF3T3 monolayers were pretreated with U0126 (30 min), scratch wounded and stained for SLK and Rac1 (I and J). SLK and Rac1 failed to be recruited to the leading edge in SYF cells. Scale bar 10μ

Cell spreading and scratch wounding of fibroblast monolayers results in the recruitment of a proportion of SLK protein at the cell periphery or the leading edge, respectively (figure 1 and [13]). Microtubule dynamics and stability have been shown to be regulated by src family kinases in various systems, including stabilization by integrin-mediated FAK signaling [33]–[36]. Therefore, we investigated whether SLK recruitment to the leading edge was also FAK/c-src dependent. Monolayers of FAK-null or SYF (src/yes/fyn triple knock-out), as well as wildtype controls, were scratch wounded and co-immunostained for SLK and Rac1. Although FAK-null cells do not migrate efficiently, SLK was detected, along with Rac1, at the leading edge (figure 7C–D). Similarly, SLK and Rac1 were recruited to ruffles and the leading edge in U0126-treated cells (figure 7E and F). However, little or no SLK could be detected at the leading edge of wounded SYF monolayers (figure 7G–H). Similarly, Rac1 distribution was impaired in those cells. The distribution of both Rac1 and SLK was restored when c-src was re-expressed in SYF cells, suggesting that c-src expression is sufficient to recruit SLK at the leading edge in migrating cells.

## Discussion

Our previous studies have shown that SLK co-localizes with vinculin and Rac1 in the membrane ruffles and lamellipodia of spreading fibroblasts [13]. In addition, we have shown that SLK can indirectly associate with the microtubule network and mediate actin stress fiber dissolution in a Rac1-dependent manner [13]. Here, we show that SLK can be co-localized with paxillin, α-tubulin and Rac1 at the leading edge of migrating cells and that its activity is upregulated by scratch wounding of fibroblast monolayers. A reduction in SLK levels or activity negatively affects cell migration and microtubule-dependent focal adhesion turnover, suggesting that SLK is required for cell motility. Cell migration by monolayer wounding stimulates SLK activity in a FAK/src/MAPK signaling dependent manner. In addition, efficient recruitment of SLK to the leading edge of migrating cells required c-src.

Overall, our data suggest that SLK is an important regulator of focal adhesions/contacts dynamics. The activation and recruitment of SLK at the leading edge of migrating cells may be necessary for the destabilization of focal contacts, a process required for further protrusive activity and motility [2], [17], [23], [37]. Focal adhesion/contact disassembly would also destabilize the actin network, a phenotype previously reported to be induced by SLK overexpression [11], [13].

The microtubule network has been shown to be tightly linked to cell adhesion and motility [26], [36], [38]. It has been reported that microtubules target focal contacts to modify their characteristics, including their disassembly [21], [39]. In addition, the microtubule network has been reported to be stabilized by FAK and Rho GTPase signaling at the leading edge of migrating cells [36], [40], [41]. The association of SLK with the microtubule and its requirement for efficient focal adhesion turnover suggests that it is a novel microtubule-associated signal required for cell migration.

Interestingly, inhibition of src family kinases by PP2 results in an upregulation of SLK activity in confluent unscratched monolayers, suggesting that src family kinases negatively regulates SLK (see figure 6) which is alleviated by PP2 treatment. Consequently, SLK cannot be further activated by wounding. Supporting this, we have previously shown that SLK is not tyrosine phosphorylated and that v-src overexpression results in SLK downregulation through casein kinase II [32]. Although it cannot be activated, SLK can still be recruited to the leading edge of FAK-null cells. However, SLK recruitment in these structures is impaired in SYF cells (see figure 6).

**Figure 8:**
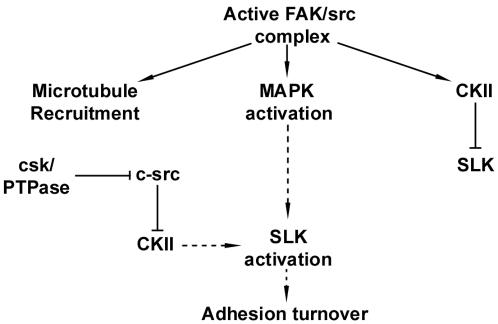
Model for SLK activation and recruitment at the leading edge. A proportion of SLK is microtubule-associated, likely through a microtubule-binding protein. Following activation of the FAK/c-src complex, the microtubule network can be recruited as well as activation of a MAPK cascade. Signaling through CKII by active src may keep SLK inactive (32) until src inactivation can occur through csk and other phosphatases. The combined MAPK activation and CKII downregulation may contribute to SLK activation. It remains to be elucidated whether SLK is recruited prior to its activation or whether CKII downregulation is required for MAPK-mediated SLK activation. This cascade would ultimately result in adhesion turnover by destabilization of the actin network or focal contacts/adhesions through an unknown mechanism.

One possibility is that the initial c-src recruitment and activation during focal contact assembly [42]–[46] recruits the microtubule network and SLK. Our previous results show that src can activate CKII, leading to direct downregulation of SLK (32). Therefore, it is possible that SLK is kept inactive until src family kinases have been downregulated. Following the subsequent inactivation of c-src through csk or protein phosphatases [47]–[49], SLK activity can be upregulated through a MAPK pathway (figure 8). As the recruitment of SLK to the leading edge occurs in FAK-null but not in SYF cells, it is likely that microtubule recruitment through c-src is FAK independent. One possibility is that, in FAK(-/-) cells, some aspects of the Pyk2/c-src signaling complex can compensate for the loss of FAK in the recruitment of SLK [4], [50]. However, Pyk2 cannot substitute for FAK which appears to be necessary for SLK activation, through a MAP kinase-dependent pathway [31].

Overall our data show that SLK is activated by FAK/c-src/MAPK signaling during cell migration. Its recruitment to the leading edge in a c-src-dependent manner is required for focal adhesion turnover. Whether SLK activation occurs prior to recruitment or following c-src downregulation is still unclear. Similarly, whether it is mediated by direct MAPK phosphorylation or other MAPK-dependent events remains to be elucidated. Similarly, it is not known whether members of the Rho family of GTPases impinge on SLK activation or recruitment, or on its ability to destabilize actin. The identification of SLK substrates or downstream signaling systems will further our understanding on the role of SLK in cell migration.

## Materials and Methods

### Cell culture and migration assays

MEF 3T3, wildtype, FAK(-/-) and SYF (src/fyn/yes triple mutant) cells were all maintained in Dulbecco's modified MEM (DMEM, Gibco) supplemented with 10% fetal bovine serum (FBS, Gibco), 2 mM L-glutamine (Gibco) and penicillin G (200 U ml^−1^, Gibco) in a humidified 37°C incubator at 5% CO_2_. For migration assays, MEF 3T3 cells were either infected with adenovirus or treated with control or SLK siRNAs and serum starved overnight (DMEM+0.5% FBS). Cells (1–3×10^4^ ) were then resuspended in DMEM containing 0.5% BSA and added to the top of a Boyden transwell migration chamber pre-coated with fibronectin (10 µg ml^−1^) and allowed to migrate for 3–6 hours. Residual cells were removed from the top of the chamber and the filter was rinsed in PBS, fixed in 4% PFA for 10 minutes and stained with DAPI (0.5 µg ml^−1^, Sigma). The cells that migrated to the underside of the filter were enumerated from 5 to 10 random fields using DAPI fluorescence. Cell counts were performed in triplicate for three independent experiments. Representative experiments are shown. For wound closure, confluent monolayers were scratched with a pipet tip and the % closure was evaluated after 12 hours as the average residual distance between the two migrating front over the initial distance at time zero. Ten independent measurements were recorded along the wound.

Scratch wound induced migration was performed as described [19]. Briefly, MEF 3T3 cells were plated on fibronectin-coated (10 µg ml^−1^) dishes and serum-starved confluent monolayers were then scratched with a pipette tip until approximately 50% of the monolayer was removed. Cells were then washed with PBS, refed and collected at various time points. In some experiments, the monolayers were pre-incubated for 60 minutes with 10 µM of PP3, PP2 (EMD-Calbiochem) or U0126 (Cell Signaling) and scratched wounded in the presence of inhibitors or DMSO control. For microtubule-dependent adhesion turnover assays [25], serum-starved subconfluent MEF 3T3 cells were treated with 10 µM nocodazole for 3 hours. The cultures were then washed 4 times with serum-free medium and refed with DMEM/0.5% FBS for the duration of the time course. Cultures were harvested at different time points and surveyed for pFAK-Tyr397 (BioSource) by Western blotting.

### SiRNA Knockdown and Adenovirus infections

MEF 3T3 cells plated at a density of 1–3×10^5^ in 60 mm plates were transfected with 5 or 10 pM SLK siRNA (Dharmacon) duplex (5′- GGUUGAGAUUGACAUAUUA) using Lipofectamine 2000 transfection reagent (Gibco) according to manufacturers recommendations. In some experiments, the cells were infected with an adenoviral vector expressing an SLK suppressor hairpin RNA (psiStrike; Promega) that consisted in the same siRNA sequence. Cells were collected 48 hours post-transfection and assayed for cell migration in transwell inserts and protein expression by western blot analysis. Control siRNAs consisted of Dharmacon's non-targeting duplex. Similar results were obtained with scrambled SLK siRNAs. To monitor the effect of kinase deficient SLK on cell migration, adenoviral vectors expressing kinase inactive SLK (HA-KΔC: aa 1–373 with an ATP-binding site mutation; Lys 63–> Arg) or a control (GFP or LacZ) were used to infect MEF 3T3 cultures [13]. Cells were infected at a MOI of 10 by the addition of the adenovirus directly to the cells in 0.25% FBS-DMEM 16 hours before migration assays. Expression of the SLK constructs was confirmed by anti-HA immunoblotting. LacZ and GFP expression was confirmed by immunobloting with anti-beta-galactosidase antibody (Promega) and by epifluorescence, respectively. GFP expression was confirmed in live cells by epifluorescence.

### Antibodies and immunofluorescence

The primary antibodies used in these studies were as follows: SLK polyclonal antibodies were as described previously [15]. Phospho-GSK3β(ser 9) and GSK3β (Cell Signaling), phospho FAK (Y397) (Biosource) and total FAK (Santa Cruz) Paxillin (BD Transduction labs), α-tubulin (Sigma), Rac1 (Santa Cruz) and phosphor ERK1/2 and total Erk1/2 (Santa Cruz), Paxillin (BD Transduction labs), α-tubulin (Sigma) and Rac1 (Santa Cruz) were obtained from commercial sources. Tetramethyl rhodamine isothiocyanate (TRITC)-phalloidin was obtained from Sigma.

For immunofluorescence studies, MEF 3T3 cells were plated on coverslips coated with or fibronectin (10 µg/ml) and incubated overnight. The following day, monolayers were scratched and stained after 2–4 h. Briefly, the cells were rinsed with PBS, fixed in 4% PFA and blocked in PBS containing 5% goat or donkey serum for 20 minutes. Fresh blocking solution containing primary antibody was added and incubated for 1 h at room temperature. Antibodies were detected with either anti-mouse or anti-rabbit secondaries conjugated to either fluorescein isothiocyanate (FITC) or TRITC (Sigma). The samples were visualized with a Zeiss Axioscope100 epifluorescence microscope equipped with the appropriate filters and photographed with a digital camera (Sony Corporation HB050) using the Northern Eclipse software package.

### Western blotting, immunoprecipitation and kinase assays

Cells were lysed in RIPA buffer as previously described [15] and lysates were cleared by centrifugation at 10000 g for 2 minutes. Protein concentrations were determined using protein assay dye reagent (Biorad). Equal amounts of protein (20–40 µg) were electrophoresed on 8% polyacrylamide gels and transferred to PVDF membrane. Membranes were probed with the indicated antibodies overnight at 4°C in 5% BSA or skim milk powder in 1× TBST (50 mM Tris pH 7.4, 150 mM NaCl, 0.05 Tween 20). Target proteins were detected with horseradish peroxidase coupled secondary antibodies combined with chemiluminescence (Perkin Elmer) and exposure to X-ray film.

For immunoprecipitations, 300–400 µg of protein lysate was immunoprecipitated with 1–2 µg of antibody and 20 µl of protein A sepharose (Pharmacia) for 2–12 hours. Immune complexes were recovered by centrifugation and washed with NETN buffer (20 mM Tris-HCl pH 8.0, 1 mM EDTA, 150 mM NaCl, 0.5% Nonidet P-40) and subjected to SDS-polyacrylamide gel electrophoresis (PAGE) or kinase assay. *In vitro* SLK kinase assays were performed following SLK immunoprecipitation as described previously [15]. Kinase reactions were stopped by the addition of 7 µl of 4× sodium dodecyl sulfate (SDS) sample buffer and electrophoresed on 8% SDS-PAGE. The gels were transferred to PVDF membranes and subjected to autoradiography followed by western blotting with anti-SLK antibody.

## References

[1] Mitra SK, Schlaepfer DD (2006). Integrin-regulated FAK-Src signaling in normal and cancer cells.. Curr Opin Cell Biol.

[2] Webb DJ, Parsons JT, Horwitz AF (2002). Adhesion assembly, disassembly and turnover in migrating cells – over and over and over again.. Nat Cell Biol.

[3] Brown MC, Turner CE (2004). Paxillin: adapting to change.. Physiol Rev.

[4] Sieg DJ, Hauck CR, Schlaepfer DD (1999). Required role of focal adhesion kinase (FAK) for integrin-stimulated cell migration.. J Cell Science.

[5] Hamadi A, Bouali M, Dontenwill M, Stoeckel H, Takeda K (2005). Regulation of focal adhesion dynamics and disassembly by phosphorylation of FAK at tyrosine 397.. J Cell Sci.

[6] Ren XD, Kiosses WB, Sieg DJ, Otey CA, Schlaepfer DD (2000). Focal adhesion kinase suppresses Rho activity to promote focal adhesion turnover.. J Cell Sci.

[7] Webb DJ, Donais K, Whitmore LA, Thomas SM, Turner CE (2004). FAK-Src signalling through paxillin, ERK and MLCK regulates adhesion disassembly.. Nat Cell Biol.

[8] Kaplan KB, Bibbins KB, Swedlow JR, Arnaud M, Morgan DO (1994). Association of the amino-terminal half of c-Src with focal adhesions alters their properties and is regulated by phosphorylation of tyrosine 527.. Embo J.

[9] Fincham VJ, Frame MC (1998). The catalytic activity of Src is dispensable for translocation to focal adhesions but controls the turnover of these structures during cell motility.. Embo J.

[10] Schaar DG, Varia MR, Elkabes S, Ramakrishnan L, Dreyfus CF (1996). The identification of a novel cDNA preferentially expressed in the olfactory-limbic system of the adult rat.. Brain Res.

[11] Sabourin LA, Tamai K, Seale P, Wagner J, Rudnicki MA (2000). Caspase 3 cleavage of the Ste20-related kinase SLK releases and activates an apoptosis-inducing kinase domain and an actin-disassembling region.. Mol Cell Biol.

[12] Sabourin LA, Rudnicki MA (1999). Induction of apoptosis by SLK, a Ste20-related kinase.. Oncogene.

[13] Wagner S, Flood TA, O'Reilly P, Hume K, Sabourin LA (2002). Association of the Ste20-like kinase (SLK) with the microtubule. Role in Rac1-mediated regulation of actin dynamics during cell adhesion and spreading.. J Biol Chem.

[14] Storbeck CJ, Daniel K, Zhang YH, Lunde J, Scime A (2004). Ste20-like kinase SLK displays myofiber type specificity and is involved in C2C12 myoblast differentiation.. Muscle Nerve.

[15] O'Reilly PG, Wagner S, Franks DJ, Cailliau K, Browaeys E (2005). The Ste20-like kinase SLK is required for cell cycle progression through G2.. J Biol Chem.

[16] Hao W, Takano T, Guillemette J, Papillon J, Ren G (2006). Induction of apoptosis by the Ste20-like kinase SLK, a germinal center kinase that activates apoptosis signal-regulating kinase and p38.. J Biol Chem.

[17] Schlaepfer DD, Mitra SK (2004). Multiple connections link FAK to cell motility and invasion.. Curr Opin Genet Dev.

[18] Schlaepfer DD, Hauck CR, Sieg DJ (1999). Signaling through focal adhesion kinase.. Prog Biophys Mol Biol.

[19] Etienne-Manneville S, Hall A (2001). Integrin-mediated activation of Cdc42 controls cell polarity in migrating astrocytes through PKCzeta.. Cell.

[20] Etienne-Manneville S, Hall A (2003). Cdc42 regulates GSK-3beta and adenomatous polyposis coli to control cell polarity.. Nature.

[21] Kaverina I, Krylyshkina O, Small JV (1999). Microtubule targeting of substrate contacts promotes their relaxation and dissociation.. J Cell Biol.

[22] Brunton VG, Avizienyte E, Fincham VJ, Serrels B, Metcalf CA (2005). Identification of Src-specific phosphorylation site on focal adhesion kinase: dissection of the role of Src SH2 and catalytic functions and their consequences for tumor cell behavior.. Cancer Res.

[23] Schwartz MA, Horwitz AR (2006). Integrating adhesion, protrusion, and contraction during cell migration.. Cell.

[24] Kaverina I, Krylyshkina O, Small JV (2002). Regulation of substrate adhesion dynamics during cell motility.. Inter J Biochem Cell Biol.

[25] Ezratty EJ, Partridge MA, Gundersen GG (2005). Microtubule-induced focal adhesion disassembly is mediated by dynamin and focal adhesion kinase.. Nat Cell Biol.

[26] Bershadsky A, Chausovsky A, Becker E, Lyubimova A, Geiger B (1996). Involvement of microtubules in the control of adhesion-dependent signal transduction.. Curr Biol.

[27] Brunton VG, MacPherson IR, Frame MC (2004). Cell adhesion receptors, tyrosine kinases and actin modulators: a complex three-way circuitry.. Biochim Biophys Acta.

[28] Mitra SK, Hanson DA, Schlaepfer DD (2005). Focal adhesion kinase: in command and control of cell motility.. Nat Rev Mol Cell Biol.

[29] Schaller MD, Parsons JT (1994). Focal adhesion kinase and associated proteins.. Curr Opin Cell Biol.

[30] Schaller MD (2001). Paxillin: a focal adhesion-associated adaptor protein.. Oncogene.

[31] Huang C, Jacobson K, Schaller MD (2004). MAP kinases and cell migration.. J Cell Sci.

[32] Chaar Z, O'Reilly P, Gelman I, Sabourin LA (2006). v-Src-dependent down-regulation of the Ste20-like kinase SLK by casein kinase II.. J Biol Chem.

[33] Simon JR, Graff RD, Maness PF (1998). Microtubule dynamics in a cytosolic extract of fetal rat brain.. J Neurocytol.

[34] Laurent CE, Delfino FJ, Cheng HY, Smithgall TE (2004). The Human c-Fes Tyrosine Kinase Binds Tubulin and Microtubules through Separate Domains and Promotes Microtubule Assembly 10.1128/MCB.24.21.9351-9358.2004.. Mol Cell Biol.

[35] Sulimenko V, Draberova E, Sulimenko T, Macurek L, Richterova V (2006). Regulation of Microtubule Formation in Activated Mast Cells by Complexes of {gamma}-Tubulin with Fyn and Syk Kinases.. J Immunol.

[36] Palazzo AF, Eng CH, Schlaepfer DD, Marcantonio EE, Gundersen GG (2004). Localized stabilization of microtubules by integrin- and FAK-facilitated Rho signaling.. Science.

[37] Small JV, Stradal T, Vignal E, Rottner K (2002). The lamellipodium: where motility begins.. Trends Cell Biol.

[38] Enomoto T (1996). Microtubule disruption induces the formation of actin stress fibers and focal adhesions in cultured cells: possible involvement of the rho signal cascade.. Cell Struct Funct.

[39] Kaverina I, Rottner K, Small JV (1998). Targeting, capture, and stabilization of microtubules at early focal adhesions.. J Cell Biol.

[40] Wittmann T, Bokoch GM, Waterman-Storer CM (2003). Regulation of leading edge microtubule and actin dynamics downstream of Rac1.. J Cell Biol.

[41] Cook TA, Nagasaki T, Gundersen GG (1998). Rho guanosine triphosphatase mediates the selective stabilization of microtubules induced by lysophosphatidic acid.. J Cell Biol.

[42] Arthur WT, Petch LA, Burridge K (2000). Integrin engagement suppresses RhoA activity via a c-Src-dependent mechanism.. Curr Biol.

[43] Arias-Salgado EG, Lizano S, Sarkar S, Burgge JS, Ginsberg MH (2003). Src kinase activation by direct interaction with the integrin {beta} cytoplasmic domain.. Proc Natl Acad Sci U S A.

[44] Schaller MD, Hildebrand JD, Parsons JT (1999). Complex formation with focal adhesion kinase; a mechanism to regulate activity and subcellular localization of Src kinases.. Molec Biol Cell.

[45] Schaller MD, Hildebrand JD, Shannon JD, Fox JW, Vines RR (1994). Autophosphorylation of the focal adhesion kinase, pp125FAK, directs SH2-dependent binding of pp60src.. Mol Biol Cell.

[46] Schlaepfer DD, Broome MA, Hunter T (1997). Fibronectin-stimulated signaling from a focal adhesion kinase-c-Src complex: involvement of the Grb2, p130 Cas , and Nck adaptor proteins.. Mol Cell Biol.

[47] Rengifo-Cam W, Konishi A, Morishita N, Matsuoka H, Yamori T (2004). Csk defines the ability of integrin-mediated cell adhesion and migration in human colon cancer cells: implication for a potential role in cancer metastasis.. Oncogene.

[48] McGarrigle D, Shan D, Yang S, Huang XY (2006). Role of tyrosine kinase Csk in G protein-coupled receptor- and receptor tyrosine kinase-induced fibroblast cell migration.. J Biol Chem.

[49] Angers-Loustau A, Cote JF, Charest A, Dowbenko D, Spencer S (1999). Protein tyrosine phosphatase-PEST regulates focal adhesion disassembly, migration and cytokinesis in fibroblasts.. J Cell Biol.

[50] Sieg DJ, Ilic D, Jones KC, Damsky CH, Hunter T (1998). Pyk2 and Src-family protein-tyrosine kinases compensate for the loss of FAK in fibronectin-stimulated signaling events but Pyk2 does not fully function to enhance FAK- cell migration.. Embo J.

